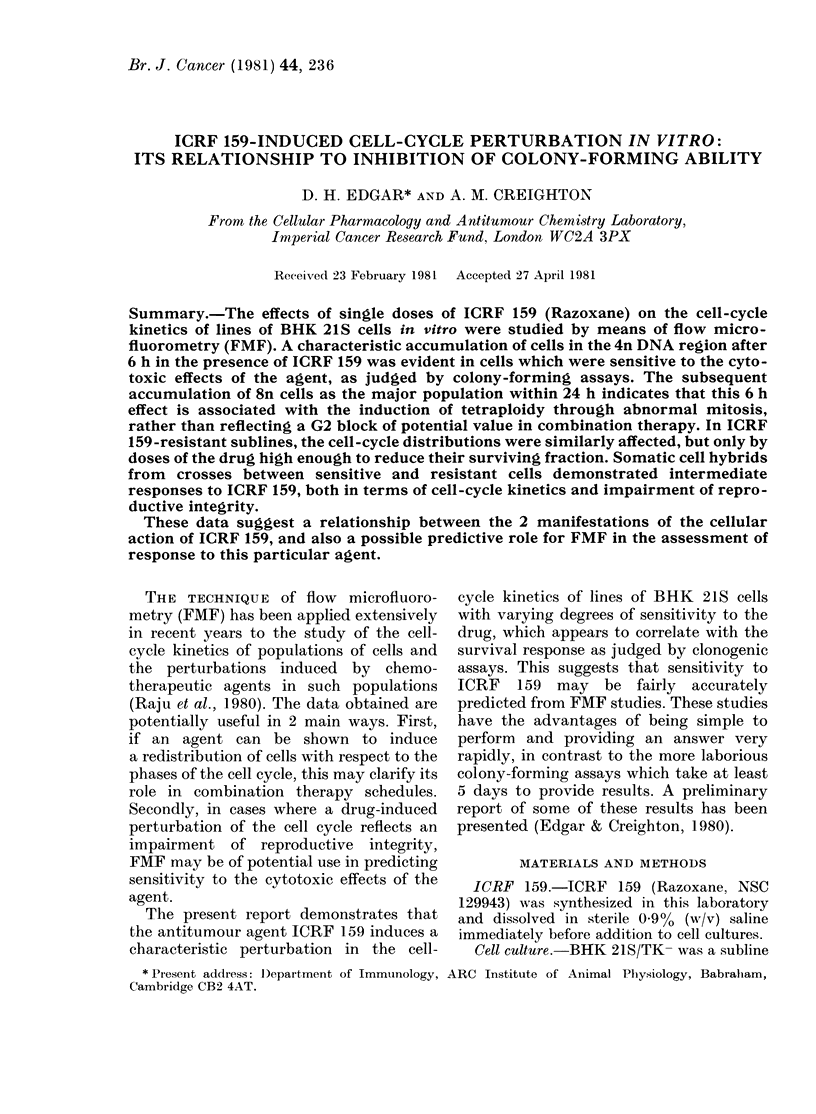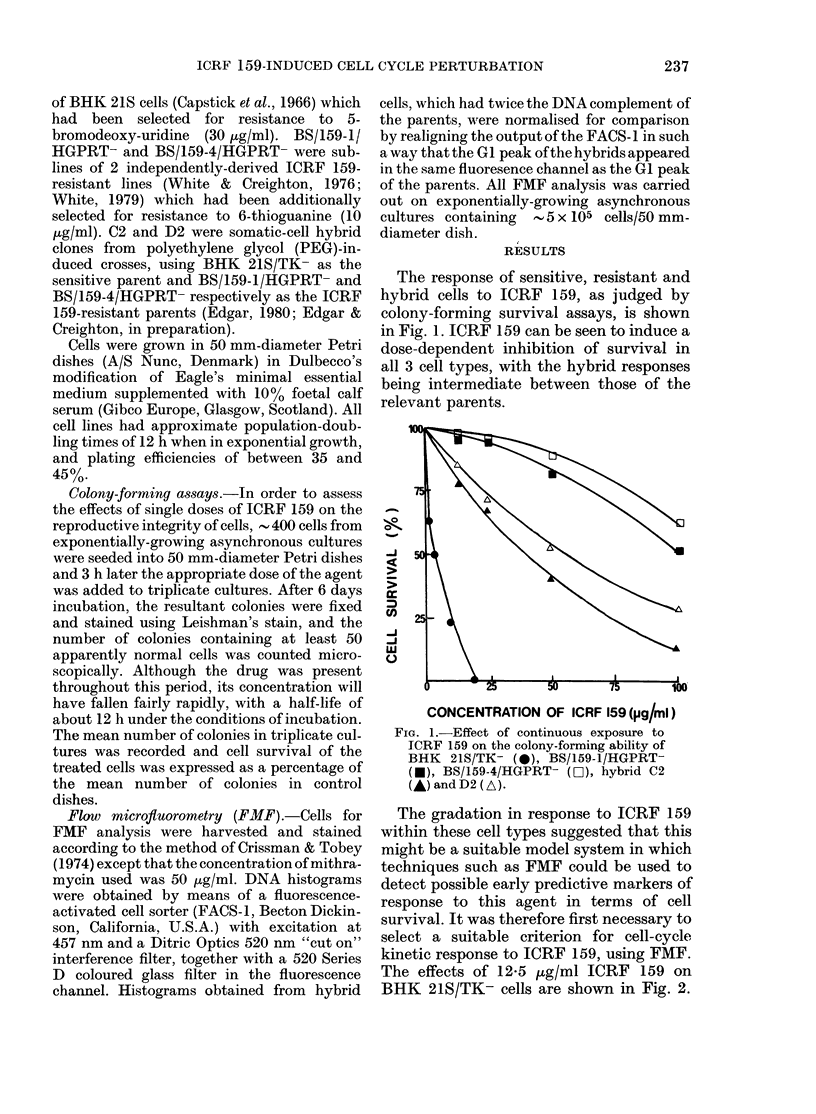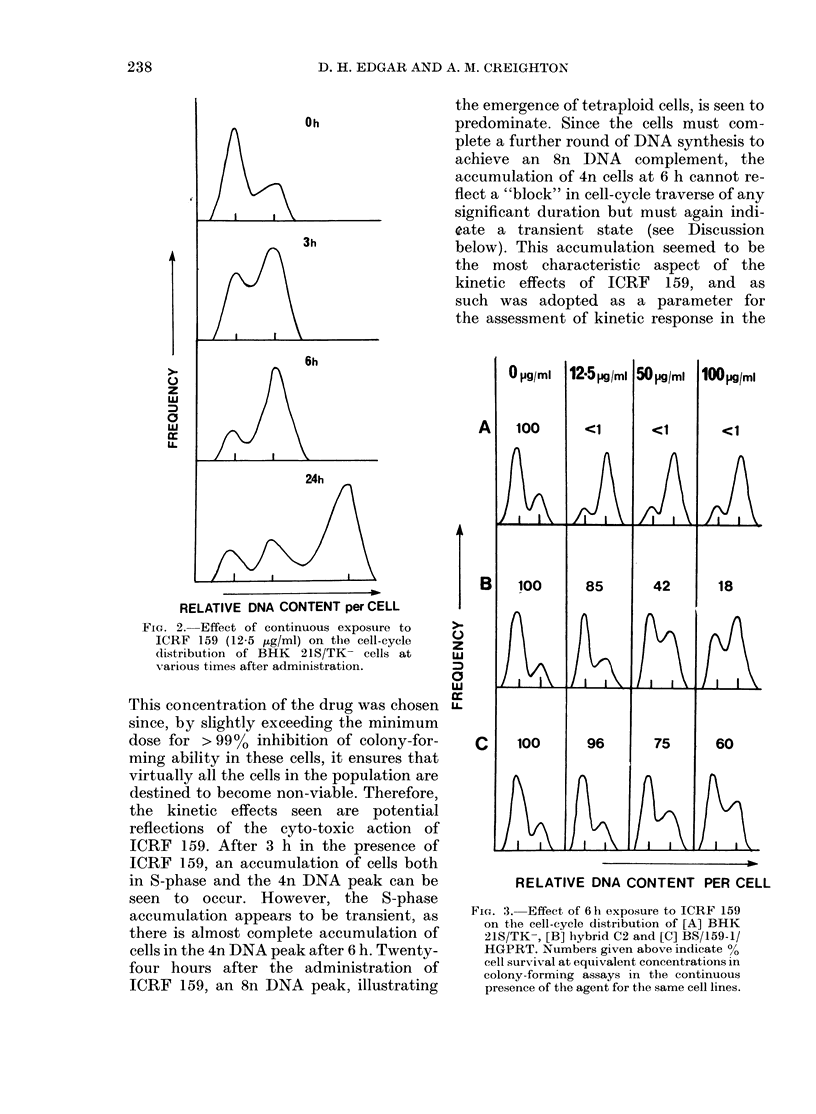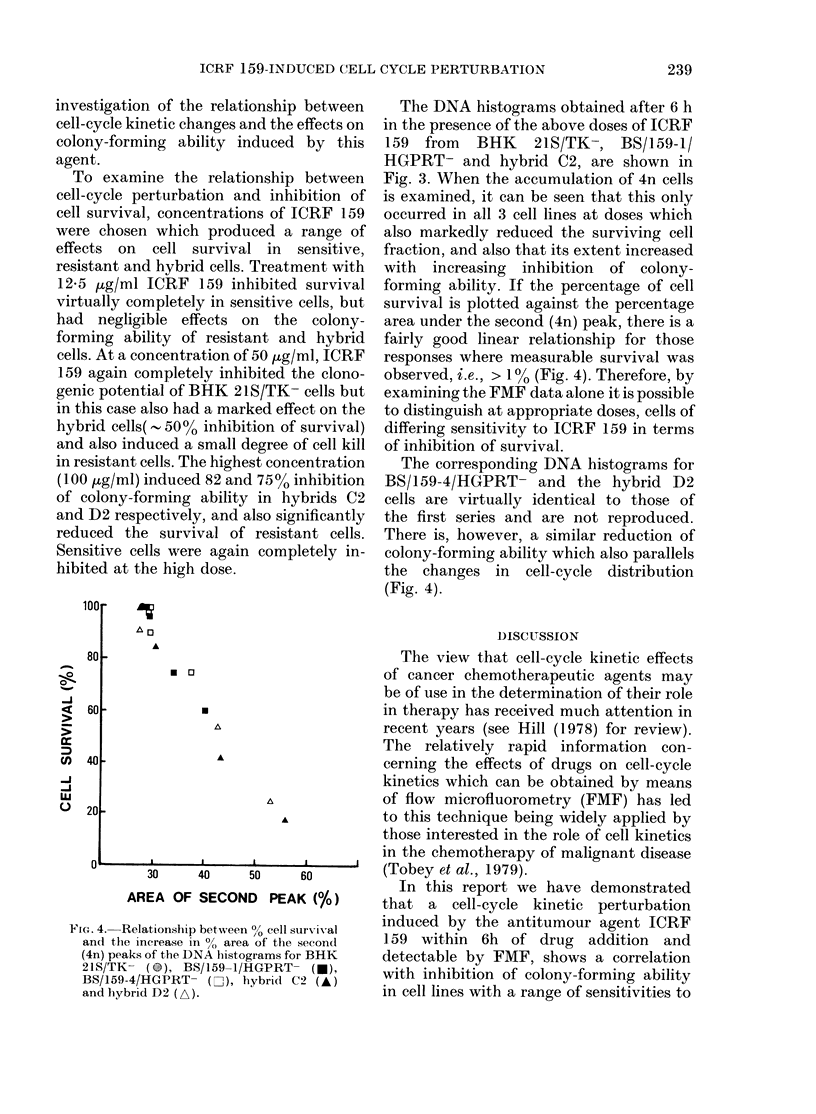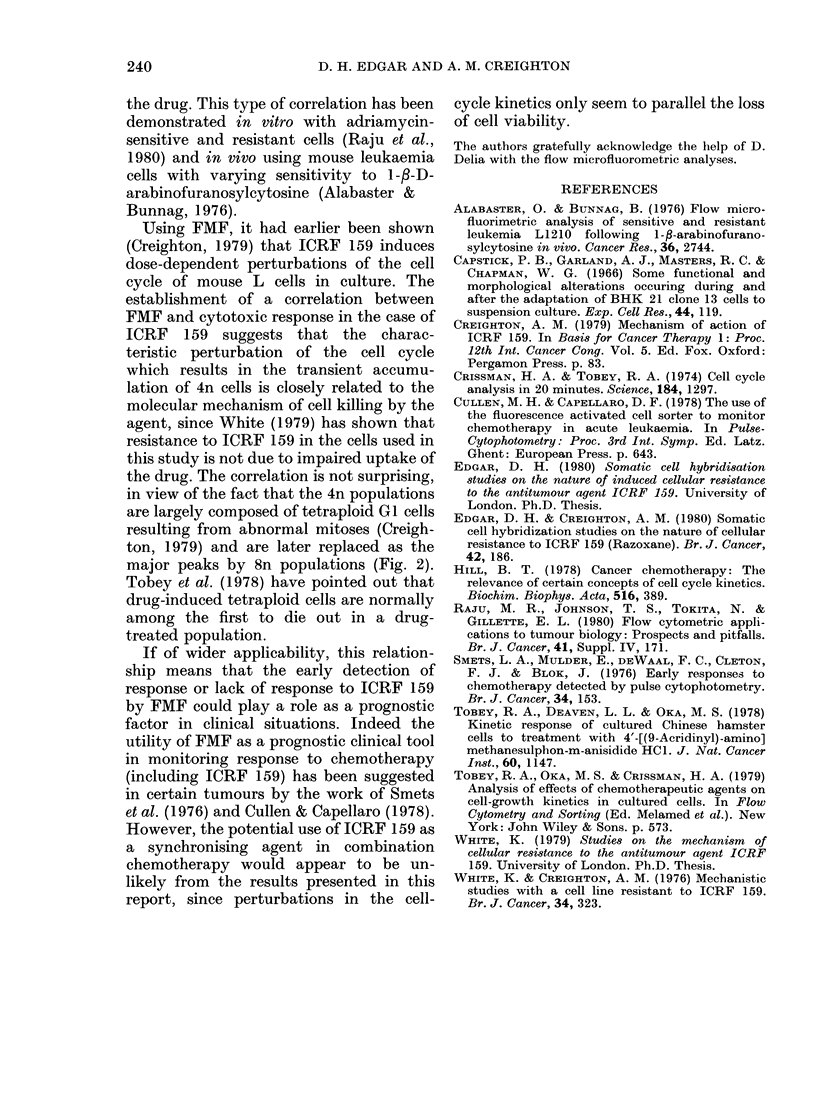# ICRF 159-induced cell-cycle perturbation in vitro: its relationship to inhibition of colony-forming ability.

**DOI:** 10.1038/bjc.1981.174

**Published:** 1981-08

**Authors:** D. H. Edgar, A. M. Creighton

## Abstract

The effects of single doses of ICRF 159 (Razoxane) on the cell-cycle kinetics of lines of BKH 21S cells in vitro were studied by means of flow micro-fluorometry (FMF). A characteristic accumulation of cells in the 4n DNA region after 6 h in the presence of ICRF 159 was evident in cells which were sensitive to the cytotoxic effects of the agents, as judged by colony-forming assays. The subsequent accumulation of 8n cells as the major population within 24 h indicates that this 6 h effect is associated with the induction of tetraploidy through abnormal mitosis, rather than reflecting a G2 block of potential value in combination therapy. In ICRF 159-resistant sublines, the cell-cycle distributions were similarly affected, but only the doses of the drug high enough to reduce their surviving fraction. Somatic cell hybrids from crosses between sensitive and resistant cells demonstrated intermediate responses in ICRF 159, both in terms of cell-cycle kinetics and impairment of reproductive integrity. These data suggest a relationship between the 2 manifestations of the cellular action of ICRF 159, and also a possible predictive role for FMF in the assessment of response to this particular agent.


*Br. J. Cancer* (1981) **44**, 236

### ICRF 159-INDUCED CELL-CYCLE PERTURBATION *IN VITRO*: ITS RELATIONSHIP TO INHIBITION OF COLONY-FORMING ABILITY

D. H. EDGAR* AND A. M. CREIGHTON

*From the Cellular Pharmacology and Antitumour Chemistry Laboratory, Imperial Cancer Research Fund, London WC2A 3PX*

Received 23 February 1981 Accepted 27 April 1981

**Summary.—The effects of single doses of ICRF 159 (Razoxane) on the cell-cycle kinetics of lines of BHK 21S cells *in vitro* were studied by means of flow microfluorometry (FMF). A characteristic accumulation of cells in the 4n DNA region after 6 h in the presence of ICRF 159 was evident in cells which were sensitive to the cytotoxic effects of the agent, as judged by colony-forming assays. The subsequent accumulation of 8n cells as the major population within 24 h indicates that this 6 h effect is associated with the induction of tetraploidy through abnormal mitosis, rather than reflecting a G2 block of potential value in combination therapy. In ICRF 159-resistant sublines, the cell-cycle distributions were similarly affected, but only by doses of the drug high enough to reduce their surviving fraction. Somatic cell hybrids from crosses between sensitive and resistant cells demonstrated intermediate responses to ICRF 159, both in terms of cell-cycle kinetics and impairment of reproductive integrity.**

**These data suggest a relationship between the 2 manifestations of the cellular action of ICRF 159, and also a possible predictive role for FMF in the assessment of response to this particular agent.**

THE TECHNIQUE of flow microfluorometry (FMF) has been applied extensively in recent years to the study of the cell-cycle kinetics of populations of cells and the perturbations induced by chemotherapeutic agents in such populations (Raju *et al.*, 1980). The data obtained are potentially useful in 2 main ways. First, if an agent can be shown to induce a redistribution of cells with respect to the phases of the cell cycle, this may clarify its role in combination therapy schedules. Secondly, in cases where a drug-induced perturbation of the cell cycle reflects an impairment of reproductive integrity, FMF may be of potential use in predicting sensitivity to the cytotoxic effects of the agent.

The present report demonstrates that the antitumour agent ICRF 159 induces a characteristic perturbation in the cell-cycle kinetics of lines of BHK 21S cells with varying degrees of sensitivity to the drug, which appears to correlate with the survival response as judged by clonogenic assays. This suggests that sensitivity to ICRF 159 may be fairly accurately predicted from FMF studies. These studies have the advantages of being simple to perform and providing an answer very rapidly, in contrast to the more laborious colony-forming assays which take at least 5 days to provide results. A preliminary report of some of these results has been presented (Edgar & Creighton, 1980).

### MATERIALS AND METHODS

*ICRF* 159.—ICRF 159 (Razoxane, NSC 129943) was synthesized in this laboratory and dissolved in sterile 0·9% (w/v) saline immediately before addition to cell cultures.

*Cell culture.*—BHK 21S/TK$^-$ was a subline

* Present address: Department of Immunology, ARC Institute of Animal Physiology, Babraham, Cambridge CB2 4AT.

of BHK 21S cells (Capstick *et al.*, 1966) which had been selected for resistance to 5-bromodeoxy-uridine (30 μg/ml). BS/159-1/HGPRT$^-$ and BS/159-4/HGPRT$^-$ were sub-lines of 2 independently-derived ICRF 159-resistant lines (White & Creighton, 1976; White, 1979) which had been additionally selected for resistance to 6-thioguanine (10 μg/ml). C2 and D2 were somatic-cell hybrid clones from polyethylene glycol (PEG)-induced crosses, using BHK 21S/TK$^-$ as the sensitive parent and BS/159-1/HGPRT$^-$ and BS/159-4/HGPRT$^-$ respectively as the ICRF 159-resistant parents (Edgar, 1980; Edgar & Creighton, in preparation).

Cells were grown in 50 mm-diameter Petri dishes (A/S Nunc, Denmark) in Dulbecco's modification of Eagle's minimal essential medium supplemented with 10% foetal calf serum (Gibco Europe, Glasgow, Scotland). All cell lines had approximate population-doubling times of 12 h when in exponential growth, and plating efficiencies of between 35 and 45%.

*Colony-forming assays.*—In order to assess the effects of single doses of ICRF 159 on the reproductive integrity of cells, ~400 cells from exponentially-growing asynchronous cultures were seeded into 50 mm-diameter Petri dishes and 3 h later the appropriate dose of the agent was added to triplicate cultures. After 6 days incubation, the resultant colonies were fixed and stained using Leishman's stain, and the number of colonies containing at least 50 apparently normal cells was counted microscopically. Although the drug was present throughout this period, its concentration will have fallen fairly rapidly, with a half-life of about 12 h under the conditions of incubation. The mean number of colonies in triplicate cultures was recorded and cell survival of the treated cells was expressed as a percentage of the mean number of colonies in control dishes.

*Flow microfluorometry (FMF).*—Cells for FMF analysis were harvested and stained according to the method of Crissman & Tobey (1974) except that the concentration of mithramycin used was 50 μg/ml. DNA histograms were obtained by means of a fluorescence-activated cell sorter (FACS-1, Becton Dickinson, California, U.S.A.) with excitation at 457 nm and a Ditric Optics 520 nm "cut on" interference filter, together with a 520 Series D coloured glass filter in the fluorescence channel. Histograms obtained from hybrid cells, which had twice the DNA complement of the parents, were normalised for comparison by realigning the output of the FACS-1 in such a way that the G1 peak of the hybrids appeared in the same fluoresence channel as the G1 peak of the parents. All FMF analysis was carried out on exponentially-growing asynchronous cultures containing ~$5 \times 10^5$ cells/50 mm-diameter dish.

### RESULTS

The response of sensitive, resistant and hybrid cells to ICRF 159, as judged by colony-forming survival assays, is shown in Fig. 1. ICRF 159 can be seen to induce a dose-dependent inhibition of survival in all 3 cell types, with the hybrid responses being intermediate between those of the relevant parents.

FIG. 1.—Effect of continuous exposure to ICRF 159 on the colony-forming ability of BHK 21S/TK$^-$ (●), BS/159-1/HGPRT$^-$ (■), BS/159-4/HGPRT$^-$ (□), hybrid C2 (▲) and D2 (△).

The gradation in response to ICRF 159 within these cell types suggested that this might be a suitable model system in which techniques such as FMF could be used to detect possible early predictive markers of response to this agent in terms of cell survival. It was therefore first necessary to select a suitable criterion for cell-cycle kinetic response to ICRF 159, using FMF. The effects of 12·5 μg/ml ICRF 159 on BHK 21S/TK$^-$ cells are shown in Fig. 2.

FIG. 2.—Effect of continuous exposure to ICRF 159 (12·5 μg/ml) on the cell-cycle distribution of BHK 21S/TK⁻ cells at various times after administration.

This concentration of the drug was chosen since, by slightly exceeding the minimum dose for >99% inhibition of colony-forming ability in these cells, it ensures that virtually all the cells in the population are destined to become non-viable. Therefore, the kinetic effects seen are potential reflections of the cyto-toxic action of ICRF 159. After 3 h in the presence of ICRF 159, an accumulation of cells both in S-phase and the 4n DNA peak can be seen to occur. However, the S-phase accumulation appears to be transient, as there is almost complete accumulation of cells in the 4n DNA peak after 6 h. Twenty-four hours after the administration of ICRF 159, an 8n DNA peak, illustrating the emergence of tetraploid cells, is seen to predominate. Since the cells must complete a further round of DNA synthesis to achieve an 8n DNA complement, the accumulation of 4n cells at 6 h cannot reflect a "block" in cell-cycle traverse of any significant duration but must again indicate a transient state (see Discussion below). This accumulation seemed to be the most characteristic aspect of the kinetic effects of ICRF 159, and as such was adopted as a parameter for the assessment of kinetic response in the

FIG. 3.—Effect of 6 h exposure to ICRF 159 on the cell-cycle distribution of [A] BHK 21S/TK⁻, [B] hybrid C2 and [C] BS/159-1/HGPRT. Numbers given above indicate % cell survival at equivalent concentrations in colony-forming assays in the continuous presence of the agent for the same cell lines.

investigation of the relationship between cell-cycle kinetic changes and the effects on colony-forming ability induced by this agent.

To examine the relationship between cell-cycle perturbation and inhibition of cell survival, concentrations of ICRF 159 were chosen which produced a range of effects on cell survival in sensitive, resistant and hybrid cells. Treatment with 12·5 μg/ml ICRF 159 inhibited survival virtually completely in sensitive cells, but had negligible effects on the colony-forming ability of resistant and hybrid cells. At a concentration of 50 μg/ml, ICRF 159 again completely inhibited the clonogenic potential of BHK 21S/TK⁻ cells but in this case also had a marked effect on the hybrid cells( ~50% inhibition of survival) and also induced a small degree of cell kill in resistant cells. The highest concentration (100 μg/ml) induced 82 and 75% inhibition of colony-forming ability in hybrids C2 and D2 respectively, and also significantly reduced the survival of resistant cells. Sensitive cells were again completely inhibited at the high dose.

FIG. 4.—Relationship between % cell survival and the increase in % area of the second (4n) peaks of the DNA histograms for BHK 21S/TK⁻ (◉), BS/159-1/HGPRT⁻ (■), BS/159-4/HGPRT⁻ (□), hybrid C2 (▲) and hybrid D2 (△).

The DNA histograms obtained after 6 h in the presence of the above doses of ICRF 159 from BHK 21S/TK⁻, BS/159-1/HGPRT⁻ and hybrid C2, are shown in Fig. 3. When the accumulation of 4n cells is examined, it can be seen that this only occurred in all 3 cell lines at doses which also markedly reduced the surviving cell fraction, and also that its extent increased with increasing inhibition of colony-forming ability. If the percentage of cell survival is plotted against the percentage area under the second (4n) peak, there is a fairly good linear relationship for those responses where measurable survival was observed, *i.e.*, > 1% (Fig. 4). Therefore, by examining the FMF data alone it is possible to distinguish at appropriate doses, cells of differing sensitivity to ICRF 159 in terms of inhibition of survival.

The corresponding DNA histograms for BS/159-4/HGPRT⁻ and the hybrid D2 cells are virtually identical to those of the first series and are not reproduced. There is, however, a similar reduction of colony-forming ability which also parallels the changes in cell-cycle distribution (Fig. 4).

### DISCUSSION

The view that cell-cycle kinetic effects of cancer chemotherapeutic agents may be of use in the determination of their role in therapy has received much attention in recent years (see Hill (1978) for review). The relatively rapid information concerning the effects of drugs on cell-cycle kinetics which can be obtained by means of flow microfluorometry (FMF) has led to this technique being widely applied by those interested in the role of cell kinetics in the chemotherapy of malignant disease (Tobey *et al.*, 1979).

In this report we have demonstrated that a cell-cycle kinetic perturbation induced by the antitumour agent ICRF 159 within 6h of drug addition and detectable by FMF, shows a correlation with inhibition of colony-forming ability in cell lines with a range of sensitivities to the drug. This type of correlation has been demonstrated *in vitro* with adriamycin-sensitive and resistant cells (Raju *et al.*, 1980) and *in vivo* using mouse leukaemia cells with varying sensitivity to 1-$\beta$-D-arabinofuranosylcytosine (Alabaster & Bunnag, 1976).

Using FMF, it had earlier been shown (Creighton, 1979) that ICRF 159 induces dose-dependent perturbations of the cell cycle of mouse L cells in culture. The establishment of a correlation between FMF and cytotoxic response in the case of ICRF 159 suggests that the characteristic perturbation of the cell cycle which results in the transient accumulation of 4n cells is closely related to the molecular mechanism of cell killing by the agent, since White (1979) has shown that resistance to ICRF 159 in the cells used in this study is not due to impaired uptake of the drug. The correlation is not surprising, in view of the fact that the 4n populations are largely composed of tetraploid G1 cells resulting from abnormal mitoses (Creighton, 1979) and are later replaced as the major peaks by 8n populations (Fig. 2). Tobey *et al.* (1978) have pointed out that drug-induced tetraploid cells are normally among the first to die out in a drug-treated population.

If of wider applicability, this relationship means that the early detection of response or lack of response to ICRF 159 by FMF could play a role as a prognostic factor in clinical situations. Indeed the utility of FMF as a prognostic clinical tool in monitoring response to chemotherapy (including ICRF 159) has been suggested in certain tumours by the work of Smets *et al.* (1976) and Cullen & Capellaro (1978). However, the potential use of ICRF 159 as a synchronising agent in combination chemotherapy would appear to be unlikely from the results presented in this report, since perturbations in the cell-cycle kinetics only seem to parallel the loss of cell viability.

The authors gratefully acknowledge the help of D. Delia with the flow microfluorometric analyses.

### REFERENCES

ALABASTER, O. & BUNNAG, B. (1976) Flow microfluorimetric analysis of sensitive and resistant leukemia L1210 following 1-$\beta$-arabinofuranosylcytosine *in vivo*. *Cancer Res.*, **36**, 2744.

CAPSTICK, P. B., GARLAND, A. J., MASTERS, R. C. & CHAPMAN, W. G. (1966) Some functional and morphological alterations occuring during and after the adaptation of BHK 21 clone 13 cells to suspension culture. *Exp. Cell Res.*, **44**, 119.

CREIGHTON, A. M. (1979) Mechanism of action of ICRF 159. In *Basis for Cancer Therapy* 1: *Proc. 12th Int. Cancer Cong.* Vol. 5. Ed. Fox. Oxford: Pergamon Press. p. 83.

CRISSMAN, H. A. & TOBEY, R. A. (1974) Cell cycle analysis in 20 minutes. *Science*, **184**, 1297.

CULLEN, M. H. & CAPELLARO, D. F. (1978) The use of the fluorescence activated cell sorter to monitor chemotherapy in acute leukaemia. In *Pulse-Cytophotometry: Proc. 3rd Int. Symp.* Ed. Latz. Ghent: European Press. p. 643.

EDGAR, D. H. (1980) *Somatic cell hybridisation studies on the nature of induced cellular resistance to the antitumour agent ICRF 159.* University of London. Ph.D. Thesis.

EDGAR, D. H. & CREIGHTON, A. M. (1980) Somatic cell hybridization studies on the nature of cellular resistance to ICRF 159 (Razoxane). *Br. J. Cancer*, **42**, 186.

HILL, B. T. (1978) Cancer chemotherapy: The relevance of certain concepts of cell cycle kinetics. *Biochim. Biophys. Acta*, **516**, 389.

RAJU, M. R., JOHNSON, T. S., TOKITA, N. & GILLETTE, E. L. (1980) Flow cytometric applications to tumour biology: Prospects and pitfalls. *Br. J. Cancer*, **41**, Suppl. IV, 171.

SMETS, L. A., MULDER, E., DEWAAL, F. C., CLETON, F. J. & BLOK, J. (1976) Early responses to chemotherapy detected by pulse cytophotometry. *Br. J. Cancer*, **34**, 153.

TOBEY, R. A., DEAVEN, L. L. & OKA, M. S. (1978) Kinetic response of cultured Chinese hamster cells to treatment with 4′-[(9-Acridinyl)-amino] methanesulphon-m-anisidide HCl. *J. Nat. Cancer Inst.*, **60**, 1147.

TOBEY, R. A., OKA, M. S. & CRISSMAN, H. A. (1979) Analysis of effects of chemotherapeutic agents on cell-growth kinetics in cultured cells. In *Flow Cytometry and Sorting* (Ed. Melamed *et al.*). New York: John Wiley & Sons. p. 573.

WHITE, K. (1979) *Studies on the mechanism of cellular resistance to the antitumour agent ICRF* 159. University of London. Ph.D. Thesis.

WHITE, K. & CREIGHTON, A. M. (1976) Mechanistic studies with a cell line resistant to ICRF 159. *Br. J. Cancer*, **34**, 323.